# Identifying copepod functional groups from species functional traits

**DOI:** 10.1093/plankt/fbv096

**Published:** 2015-11-03

**Authors:** Fabio Benedetti, Stéphane Gasparini, Sakina-Dorothée Ayata

**Affiliations:** Sorbonne Universités, UPMC Univ Paris 06, INSU-CNRS, Laboratoire D'Océanographie de Villefranche, 181 Chemin du Lazaret, 06230 Villefranche-Sur-Mer, France

**Keywords:** copepods, functional trait, zooplankton, Mediterranean Sea

## Abstract

We gathered information on the functional traits of the most representative copepod species in the Mediterranean Sea. Our database includes 191 species described by 7 traits encompassing diverse ecological functions: minimal and maximal body length, trophic group, feeding type, spawning strategy, diel vertical migration and vertical habitat. Cluster analysis in the functional trait space revealed that Mediterranean copepods can be separated into groups with distinct ecological roles.

Functional traits are phenotypic characteristics of organisms that impact their fitness and are relevant to ecosystem function (Violle *et al*., 2007). For zooplankton, traits can be classified according to ecological functions—feeding, growth/reproduction, survival— and types—morphological, physiological, behavioural, life history (Litchman *et al*., 2013). As organisms have to allocate energy among ecological functions, traits relate to one another through trade-offs (Kiørboe *et al*., 2015). Litchman *et al.* (Litchman *et al*., 2013) recently advocated the implementation of a comprehensive matrix of key functional traits for zooplankton taxa. However, these functional traits have mainly been documented from laboratory experiments, thereby only encompassing a small number of species (Barnett *et al*., 2007; Saiz and Calbet, 2007; Barton *et al*., 2013; Kiørboe *et al*., 2015). For application in marine ecology, traits are needed at the species level and for the largest possible number of species, which requires the gathering of a huge amount of specific information. Such a challenge should be tackled step by step, focusing on some taxa and/or ocean basins at first. Functional traits could be used to gather species with similar traits into functional groups (i.e. sets of species with similar effects on ecosystem functions and/or similar response to environmental conditions; Gitay and Noble, 1997) or to describe functional diversity of zooplankton communities (Barnett *et al*., 2007; Vogt *et al*., 2013; Pomerleau *et al*., 2015). Identifying and describing zooplankton functional groups should then increase our understanding of zooplankton ecological roles in marine ecosystems.

In this study, we developed a trait database for the most commonly sampled and abundant Mediterranean copepod species. Copepods are abundant in marine pelagic ecosystems (e.g. Siokou-Frangou *et al*., 2010) where they constitute the main trophic link between primary producers and higher trophic levels, such as small pelagic fishes (Costalago *et al*., 2015). Copepods are also relatively well documented in terms of distribution and biology (Razouls *et al*., 2005–2015). From this trait database, the aim of this study was to estimate how many functional groups could be identified among Mediterranean copepods, to characterize them and to discuss their ecological significance.

We considered 191 copepod species (Table I) that are the most representative of the Mediterranean copepod communities, in terms of both abundance and presence (Siokou-Frangou *et al*., 2010; Mazzocchi *et al*., 2014). More details on how this list of species has been gathered are available in Supplementary data, Material S1. We used the following traits covering various types and ecological functions (Litchman *et al*., 2013), known to be ecologically meaningful (Kiørboe and Sabatini, 1994; Kiørboe, 2011; Kiørboe *et al*., 2015) and commonly used for zooplankton (Barnett *et al*., 2007; Barton *et al*., 2013; Pomerleau *et al*., 2015):

- two morphological traits which relate to many ecological traits: minimum and maximum adult body (cephalothorax) length (mm);
- one physiological trait defining the species' trophic group (Carnivore, Omnivore–Carnivore, Omnivore, Omnivore–Herbivore, Omnivore–Detritivore);
- one behavioural trait depicting feeding strategy, classified into three classes (Kiørboe, 2011): active ambush feeding, cruise feeding and filter feeding, mixed feeding (for species that can switch between the three strategies);
- one life history trait related to reproduction, defining the egg-spawning strategy (broadcast-spawner, sac-spawner);
- one behavioural trait related to diel vertical migration (DVM) behaviour, classified into four classes according to the intensity of the observed migration: Non-migrant, Weak migrant (DVM occurs within tens of metres), Strong migrant (over several hundreds of metres), Reverse migrant (for species that migrate deeper at night).

**Table I:** List of the 191 Mediterranean copepod species whose traits have been described

Functional group											
1		2		3		4		5		6	
Number	Species	Number	Species	Number	Species	Number	Species	Number	Species	Number	Species
5	*Aetideopsis armata*	**6**	*Aetideus armatus*	**1**	*Acartia clausi*	8	*Anomalocera patersoni*	69	*Diaixis pygmaea*	**45**	*Clausocalanus arcuicornis*
10	*Augaptilus longicaudatus*	**7**	*Aetideus giesbrechti*	**2**	*Acartia danae*	**12**	*Calanoides carinatus*	70	*Disco minutus*	**46**	*Clausocalanus furcatus*
11	*Augaptilus spinifrons*	29	*Candacia bispinosa*	**3**	*Acartia discaudata*	13	*Calanopia elliptica*	71	*Distioculus minor*	**47**	*Clausocalanus jobei*
27	*Candacia armata*	31	*Candacia giesbrechti*	**4**	*Acartia negligens*	**14**	*Calanus helgolandicus*	96	*Homeognathia brevis*	**50**	*Clausocalanus parapergens*
28	*Candacia bipinnata*	34	*Candacia simplex*	9	*Archescolecithrix auropecten*	15	*Calocalanus adriaticus*	113	*Monothula subtilis*	**51**	*Clausocalanus paululus*
30	*Candacia ethiopica*	35	*Candacia tenuimana*	**37**	*Centropages chierchiae*	16	*Calocalanus contractus*	118	*Neomormonilla minor*	**52**	*Clausocalanus pergens*
32	*Candacia longimana*	36	*Candacia varicans*	**38**	*Centropages furcatus*	17	*Calocalanus elegans*	**119**	*Oithona atlantica*	79	*Euchirella messinensis*
33	*Candacia norvegica*	**56**	*Corycaeus anglicus*	**39**	*Centropages hamatus*	18	*Calocalanus elongatus*	**120**	*Oithona brevicornis*	80	*Euchirella rostrata*
54	*Copilia quadrata*	**57**	*Corycaeus brehmi*	**40**	*Centropages kroyeri*	19	*Calocalanus longisetosus*	**121**	*Oithona decipiens*	81	*Euchirella truncata*
**75**	*Euchaeta acuta*	**58**	*Corycaeus clausi*	**41**	*Centropages ponticus*	20	*Calocalanus neptunus*	**122**	*Oithona linearis*	82	*Euterpina acutifrons*
**76**	*Euchaeta marina*	**59**	*Corycaeus flaccus*	**42**	*Centropages typicus*	21	*Calocalanus pavo*	**123**	*Oithona longispina*	85	*Goniopsyllus rostratus*
**77**	*Euchaeta media*	**60**	*Corycaeus furcifer*	**43**	*Centropages violaceus*	22	*Calocalanus pavoninus*	**124**	*Oithona nana*	97	*Isias clavipes*
**78**	*Euchaeta spinosa*	**61**	*Corycaeus giesbrechti*			23	*Calocalanus plumatus*	**125**	*Oithona parvula*	106	*Macrosetella gracilis*
86	*Haloptilus acutifrons*	**62**	*Corycaeus latus*			24	*Calocalanus plumulosus*	**126**	*Oithona plumifera*	107	*Mecynocera clausi*
87	*Haloptilus angusticeps*	**63**	*Corycaeus limbatus*			25	*Calocalanus styliremis*	**128**	*Oithona similis*	**109**	*Microcalanus pygmaeus*
89	*Haloptilus mucronatus*	**64**	*Corycaeus minimus*			26	*Calocalanus tenuis*	**129**	*Oithona tenuis*	**110**	*Microsetella norvegica*
90	*Haloptilus ornatus*	**65**	*Corycaeus ovalis*			**44**	*Chiridius poppei*	**130**	*Oithona vivida*	**111**	*Microsetella rosea*
91	*Haloptilus oxycephalus*	**66**	*Corycaeus speciosus*			**48**	*Clausocalanus lividus*	188	*Vettoria granulosa*	**127**	*Oithona setigera*
92	*Haloptilus tenuis*	**67**	*Corycaeus typicus*			**49**	*Clausocalanus mastigophorus*	189	*Vettoria longifurca*	**131**	*Oncaea curta*
93	*Heterorhabdus abyssalis*	72	*Euaugaptilus hecticus*			53	*Copilia mediterranea*	190	*Vettoria parva*	**132**	*Oncaea media*
95	*Heterorhabdus spinifrons*	**83**	*Farranula carinata*			55	*Copilia vitrea*			**133**	*Oncaea mediterranea*
**143**	*Paraeuchaeta hebes*	**84**	*Farranula rostrata*			**68**	*Ctenocalanus vanus*			**134**	*Oncaea ornata*
**144**	*Paraeuchaeta norvegica*	88	*Haloptilus longicornis*			**73**	*Eucalanus elongatus*			**135**	*Oncaea scottodicarloi*
147	*Phaenna spinifera*	94	*Heterorhabdus papilliger*			74	*Eucalanus hyalinus*			**136**	*Oncaea venusta*
**158**	*Sapphirina angusta*	99	*Lubbockia aculeata*			98	*Labidocera wollastoni*			**137**	*Oncaea waldemari*
**160**	*Sapphirina gemma*	100	*Lubbockia squillimana*			**101**	*Lucicutia clausi*			142	*Paracartia latisetosa*
**161**	*Sapphirina intestinata*	138	*Pachos punctatum*			**102**	*Lucicutia flavicornis*			**155**	*Pseudocalanus elongatus*
**163**	*Sapphirina metallina*	145	*Parapontella brevicornis*			**103**	*Lucicutia gaussae*			170	*Scaphocalanus curtus*
**164**	*Sapphirina nigromaculata*	153	*Pontellina plumata*			**104**	*Lucicutia gemina*			171	*Scaphocalanus invalidus*
**165**	*Sapphirina opalina*	154	*Pontellopsis villosa*			**105**	*Lucicutia ovalis*			172	*Scolecithricella abyssalis*
**166**	*Sapphirina ovatolanceolata*	**159**	*Sapphirina auronitens*			**108**	*Mesocalanus tenuicornis*			173	*Scolecithricella dentata*
**167**	*Sapphirina sali*	**162**	*Sapphirina lactens*			112	*Monacilla typica*			174	*Scolecithricella tenuiserrata*
**168**	*Sapphirina scarlata*	**169**	*Sapphirina vorax*			114	*Mormonilla phasma*			175	*Scolecithricella vittata*
						**115**	*Nannocalanus minor*			176	*Scolecithrix bradyi*
						**116**	*Neocalanus gracilis*			177	*Scolecithrix danae*
						117	*Neocalanus robustior*			178	*Spinocalanus abyssalis*
						**139**	*Paracalanus denudatus*			179	*Spinocalanus longicornis*
						**140**	*Paracalanus nanus*			183	*Triconia conifera*
						**141**	*Paracalanus parvus*			184	*Triconia dentipes*
						146	*Pareucalanus attenuatus*			185	*Triconia minuta*
						**148**	*Pleuromamma abdominalis*			186	*Triconia similis*
						**149**	*Pleuromamma borealis*			187	*Triconia umerus*
						**150**	*Pleuromamma gracilis*			191	*Xanthocalanus agilis*
						**151**	*Pleuromamma xiphias*				
						152	*Pontella mediterranea*				
						**156**	*Rhincalanus cornutus*				
						**157**	*Rhincalanus nasutus*				
						180	*Subeucalanus crassus*				
						181	*Subeucalanus monachus*				
						**182**	*Temora stylifera*				

The species are numbered by alphabetical order but gathered by functional groups, as revealed by the hierarchical clustering on the first four axes of the MCA on functional traits (see Fig. 1). The species with bold numbers are the 99 species used in the MCA space calculation.

All body sizes were obtained from Razouls *et al.* (Razouls *et al*., 2005–2015), whereas the other traits were obtained from an extensive literature review (see Supplementary data, Material S4 and Table SII for the full list of references). Additionally, to discuss the potential role of each functional group in the pelagic ecosystem, the species’ preferential depth layer was established (epi-/meso-/bathypelagic). We were able to determine at least 5 of the 7 functional traits for 171 species. The trait database for the 191 copepod species is available as Supplementary Table SII and can also be downloaded from PANGAEA (http://doi.pangaea.de/10.1594/PANGAEA.854331).

In order to identify functional groups, we performed a multiple correspondence analysis (MCA) on the trait matrix. MCA is an ordination method in reduced space for the multivariate analysis of categorical variables (Husson *et al*., 2010). The computation of the MCA functional space was performed on four traits: class of maximum body length (Size_1: 0.501.80 mm, Size_2: 1.89–2.85 mm, Size_3: 3.00–5.70 mm, Size_4: 6.10–11.0 mm), binary trophic group (Carnivore, Omnivore, Herbivore, Detritivore), feeding type and spawning strategy. Indeed, the minimum body length was highly correlated to the maximum body length (*R*^2^ = 0.866, *n* = 191) and DVM behaviour was not taken into account since it tends to be very plastic for most species, meaning that it is known to vary greatly according to the environmental fluctuations and species' ontogeny (see Pomerleau *et al*., 2015). A preliminary MCA incorporating these traits showed they had no impact in the definition of functional groups. Species for which the four traits were not fully defined were used as supplementary objects, meaning that they are associated with a group *a posteriori*, from their informed traits (see Supplementary data, Material S2 for more details on the MCA). The Euclidean distance among the 191 species in the functional space was computed using their coordinates along the four significant axes of the MCA (70.77% of the variance). Hierarchical agglomerative clustering was performed on this distance matrix using a synoptic aggregation method (Ward's; Husson *et al*., 2010). Depending on the cutting level, two, three or six clusters could be identified (Fig. 1). The first level distinguished species according to the trophic group (carnivore vs. non-carnivore). Among non-carnivore species, the second level separated broadcasters from sac-spawners. Then, each of these groups was divided into two subgroups with different size and/or feeding type. Since higher cut levels could not be clearly related to functional traits, six functional groups were retained (Tables I and II). We will now detail each functional group and discuss their ecological role in the Mediterranean pelagic food web.

**Table II:** Traits characterization of the six identified functional groups (Fig. 1)

		Functional groups						Total number of species
Functional trait	Category	Group 1	Group 2	Group 3	Group 4	Group 5	Group 6	
Class of maximum body length	Size_1 (0.50–1.80 mm)	0	12	6	16	20	32	86
	Size_2 (1.89–2.85 mm)	0	20	6	17	0	8	51
	Size_3 (3.00–5.70 mm)	30	1	0	10	0	1	42
	Size_4 (6.10–11.0 mm)	3	0	0	7	0	2	12
Trophic group	Carnivore	25	29	0	0	0	0	54
	Omnivore	0	0	6	12	11	4	33
	Omnivore–carnivore	6	1	1	0	0	0	8
	Omnivore–detritivore	0	0	0	0	0	28	28
	Omnivore–herbivore	0	0	4	36	0	11	51
	NA	2	3	1	2	9	0	17
Feeding type	Active ambush	0	16	0	0	11	1	28
	Cruise	11	5	0	3	0	15	34
	Filter	4	0	0	43	0	8	55
	Mixed	0	0	11	0	0	2	13
	NA	18	12	1	4	9	17	61
Spawning strategy	Broadcaster	7	7	11	24	0	3	52
	Sac-spawner	16	20	0	3	17	28	84
	NA	10	6	1	23	3	12	55
Type of DVM	No DVM	13	8	6	13	2	15	57
	Weak DVM	0	1	0	0	2	2	5
	Strong DVM	2	1	0	8	0	2	13
	Reverse DVM	5	12	2	15	2	14	50
	NA	13	11	4	14	14	10	66
Vertical distribution	Epipelagic (0–200 m)	10	2	11	21	7	14	65
	Epimesopelagic (0–1000 m)	11	14	0	17	7	13	62
	Epibathypelagic (0–4000 m)	11	15	0	9	2	9	46
	Mesopelagic (200–1000 m)	0	1	0	0	0	2	3
	Mesobathypelagic (200–4000 m)	1	0	1	3	2	4	11
	NA	0	1	0	0	2	1	4
Mean minimum body length (mm)		2.27	1.18	1.07	1.47	0.67	0.93	–
Mean maximum body length (mm)		4.47	2.03	1.82	2.95	1.11	1.74	–
Total number of species		33	33	12	50	20	43	191

The number of species recorded within each trait's class and within each functional group is reported. The groups are based on hierarchical clustering on the first four axes of the MCA based on four functional traits: class of maximum body length, binary trophic group, feeding type and spawning strategy. For information, the type of DVM, the vertical distribution, the average minimum body length (mm) and the average maximum body length (mm) are also indicated (in grey).

NA, not available; DVM, diel vertical migration.

**Fig. 1.**
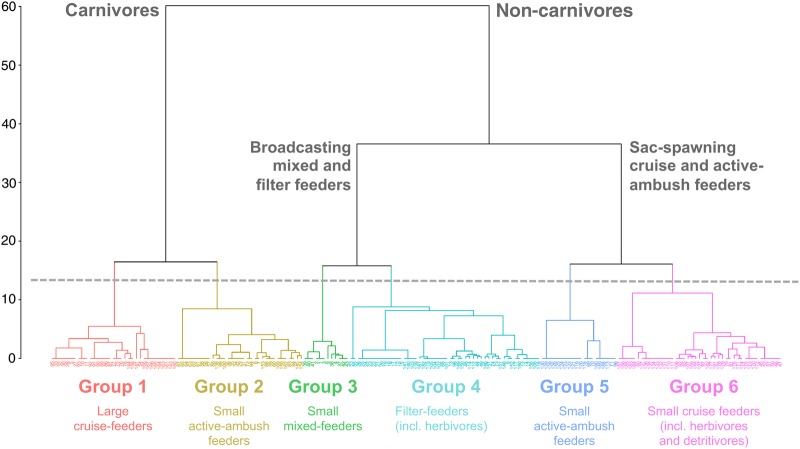
Identification of functional groups among the 191 most representative copepod species of the Mediterranean Sea from hierarchical clustering on the first four axes of the MCA based on four functional traits (class of maximum body length, binary trophic group, feeding type and spawning strategy). Depending on the cutting level, two, three or six clusters could be identified. The first level distinguished species according to trophic group (carnivore vs. non-carnivore). Among non-carnivore species, the second level discriminates broadcasters vs. sac-spawners. Then, each of these groups can be divided into two subgroups with different size and/or feeding type. Since higher cut levels could not be clearly related to functional traits, six functional groups were retained (Table II). Copepod species are indicated by numbers (Table I) .

Group 1 is composed of large carnivores that primarily feed by cruising. There is no unique reproductive strategy, though the species are mainly sac-spawners. Many species have a broad vertical distribution, ranging from the epipelagic to the bathypelagic. Representative genera are Calanoids such as *Candacia* spp., *Haloptilus* spp., *Heterorhabdus* spp., members of the Euchaetidae family and Cyclopoids of the *Sapphirina* genus. These species are known to prey on smaller copepods, as well as other zooplanktonic taxa, such as doliolids (Takahashi *et al*., 2013), larvaceans (Ohtsuka and Onbé, 1989) and even fish larvae (Yen, 1987).

Group 2 is defined by smaller carnivore species, all active ambush feeders and mainly sac-spawners, belonging to the Corycaeidae family. These are small visual predators that prey on nanozooplankton, nauplii, younger stages of copepodites through active ambush tactics (Landry *et al*., 1985), on wide depth intervals. Together, Groups 1 and 2 mainly contribute to the top-down control of mesozooplankton, including the other copepod functional groups.

Group 3 gathers a lesser number of species and consists of Calanoids of the genera *Centropages* and *Acartia*. They are small omnivorous broadcasters, but phytoplankton can become an important component of their diet. They exhibit mixed feeding strategies, depending on the available food items. *Acartia* spp. and *Centropages* spp. are generally restricted to the epipelagic and are affiliated with neritic environments (Siokou-Frangou *et al*., 2010).

Group 4 is the largest group and comprises almost all filter-feeding species, spanning all size classes, with a clear tendency towards herbivory. The species of this group for which reproductive strategy could be found were mainly broadcasters. This group contains not only small-bodied calanoids that are numerically very important in the Mediterranean epipelagic (*Clausocalanus* spp., *Calocalanus* spp., *Temora stylifera*; Mazzocchi *et al*., 2014), but also larger calanoids, some of which are strong vertical migrants, such as *Calanus helgolandicus*, *Pleuromamma* spp. or *Neocalanus* spp. (Andersen *et al*., 2001, 2004). The small surface calanoids are the target prey for larval and juvenile pelagic fish (Borme *et al*., 2013; Costalago *et al*., 2015), whereas larger calanoids are the preferential prey of Mediterranean mesopelagic fishes (Palma, 1990). Additionally, the strong calanoid migrants might play a differential key role in carbon cycling as they graze upon microalgae in the euphotic zone, and then migrate below the permanent thermocline where they excrete their lipid reserves (lipid pump hypothesis; Jónasdóttir *et al*., 2015). Therefore, Groups 3 and 4 are crucial for the transfer of energy from photoautotrophs to higher trophic levels, both in neritic (Group 3) and in oceanic environments (Group 4). Also, the latter group might comprise species that play a potentially underestimated role for the carbon flux (Jónasdóttir *et al*., 2015).

Group 5 consists essentially of *Oithona* spp. These are small active ambush-feeding omnivores that carry their eggs. It is difficult to assign a particular ecological function to such a group, since *Oithona* spp. are a major component of the global ocean's plankton, independently of environmental conditions (Gallienne and Robins, 2001). Feeding and trophic group were unknown for the other species of Group 5. Therefore, they are related to *Oithona* spp. only because of they are small (<1.8 mm) sac-spawners. Group 6 also comprised small sac-spawning omnivores, but these are mainly cruising detritivores (*Oncaea* spp., *Microsetella* spp.) or herbivores (*Clausocalanus* spp.). The former usually exhibit a wide vertical distribution, while the latter are epipelagic. *Oncaea* spp. and *Microsetella* spp. are known associates of appendicularian houses (Alldredge, 1972; Steinberg *et al*., 1994). Several calanoid species of this group (*Euchirella* spp., Scolecitrichidae) are also deep-water detritivores. Consequently (and as suggested by a higher cut level on dendrogram Fig. 1), Group 6 could be sensibly divided into two subgroups: (i) deep-water dwelling detritivores that actively participate in the recycling of particulate organic matter and (ii) small cruising grazers contributing to epipelagic secondary production.

By focusing on Mediterranean copepods, we were able to gather information on functional traits for 191 species, with at least 6 traits described for 135 species and 7 for 66 species. Using 4 of these traits, functional groups with different ecological roles were described. Although the definition of these groups was robust (similar groups were found using a K-means partitioning method, or accounting for all traits), the main limitation, as for any trait-based approach, remains the scarcity of trait descriptions at the species level. Compiling a trait database for a larger number of zooplankton species remains challenging, but the present initiative demonstrates the usefulness of this endeavour.

The present study also confirmed or revealed trade-offs among zooplankton functional traits (Litchman *et al*., 2013, Kiørboe, 2011). For instance, small carnivores and small omnivores were active ambush feeders, while large carnivores were cruise feeders. This relationship between size and feeding strategy could be explained by the differences in metabolic requirements. Indeed, whereas cruise feeders have to swim actively to encounter their prey, ambush feeders passively encounter them (Kiørboe, 2011), which requires less energy. As metabolic rates scale with body size (Kiørboe and Hirst, 2014), this difference in metabolic requirement could explain their difference in size. We also found that sac-spawners were active ambush feeders or cruise feeders, whereas broadcasters were mainly filter feeders or mixed feeders. This could be due to optimal resource allocation: egg-carrying ambush-feeding copepods have longer lifespans and lower fecundity rates than broadcasting active feeders (Kiørboe and Sabatini, 1994; Kiørboe *et al*., 2015). We also found that three quarters of the carnivorous species were sac-spawners; hence, these exhibit a higher degree of parental care and avoid predation on their own eggs. Conversely, for epipelagic omnivorous filter feeders, which are heavily preyed upon by other zooplankters and fishes, broadcasting is likely to be favoured to avoid being eaten together with the eggs, as an adaptation to the elevated mortality of ovigerous females (Kiørboe and Sabatini, 1994). Together, these results call for a better understanding of the mechanistic processes that lead to such trade-offs across traits.

The functional trait database compiled here can be used to estimate the functional diversity of zooplankton communities (Vogt *et al*., 2013; Pomerleau *et al*., 2015) and test whether traits can be related to environmental variables (Barton *et al*., 2013). Finally, the existence of several functional groups encourages the integration of more diverse planktonic assemblages in ecosystem models.

## SUPPLEMENTARY DATA

Supplementary data can be found online at http://plankt.oxfordjournals.org.

## DATA ARCHIVING

The trait database for the 191 copepod species can be downloaded from PANGAEA (http://doi.pangaea.de/10.1594/PANGAEA.854331).

## FUNDING

Financial support was provided by the EC FP7 PERSEUS Project (Grant. Agr. 287600), the MerMEx (Marine Ecosystems Response in the Mediterranean Experiment)/MISTRALS French National Program through the PlankMed action and the Climate-KIC of the European Institute of Innovation & Technology (EIT) through a PhD grant to F.B. Funding to pay the Open Access publication charges for this article was provided by the EC FP7 PERSEUS Project.

## Supplementary Material

Supplementary Data
