# Supplementary material for: Identifying copepod functional groups from species functional traits
Source: J Plankton Res. 2015 Nov 3;38(1):159–66. doi: 10.1093/plankt/fbv096 (PMC4722884; doi:10.1093/plankt/fbv096)
Supplement: Supplementary Data [file supp_38_1_159__index.html]

Identifying copepod functional groups from species functional traits — Identifying copepod functional groups from species functional traits — Supplementary Data 

# Identifying copepod functional groups from species functional traits

## Supplementary Data

Supplementary Data

- Supplementary Data - Docx file
- Supplementary Figure 1 - pdf file
- Supplementary Table 1 - xlsx file
- Supplementary Table 2 - xlsx file
