# Supplementary material for: Identifying copepod functional groups from species functional traits
Source: J Plankton Res. 2015 Nov 3;38(1):159–66. doi: 10.1093/plankt/fbv096 (PMC4722884; doi:10.1093/plankt/fbv096)
Supplement: Supplementary Data [file supp_fbv096_fbv096supp.docx]

**Identifying copepod functional groups from species functional traits**

Fabio Benedetti^1^, Stéphane Gasparini^1^, and Sakina-Dorothée Ayata^1,*^

^1^Sorbonne Universités, UPMC Univ Paris 06, INSU-CNRS, Laboratoire d'Océanographie de Villefranche, 181 Chemin du Lazaret, 06230 Villefranche-sur-mer, France

^*^Corresponding author: sakina.ayata@normalesup.org

**SUPPLEMENTARY INFORMATION**

**S1 - Functional trait database construction at the species level**

For creating a representative functional trait matrix at the species level, we listed the most commonly-sampled copepod species in the Mediterranean Sea, out of the nearly 560 species that have been recorded in this basin (Razouls et al., 2005-2015). To do so, we gathered data from the PANGAEA database and from individual sampling cruises, when available (Suppl. Table S1 and associated references in Suppl. Information S3). Records were restricted to vertical hauls, performed between the surface and 300 m depth, with plankton nets equipped with a 200 µm mesh, which is the current and standard zooplankton sampling methodology (Sameoto, 2000; UNESCO, 1968). Diel vertical migration is known to bring out deep-water zooplankton at night, however this phenomenon was shown to have little to no effect on species composition in the different implemented studies. Consequently, our species list should be representative of Mediterranean epipelagic communities (i.e. comprising the most present and abundant species), and is likely to under-estimate the contribution of smaller taxa because of the relatively coarse mesh size (Turner, 2004). In order to smoothen the effects of both temporal variability (which may promote some species according to the season) and zooplankton spatial patchiness, records were chosen so all seasons are represented, over a time period that is representative of present-day environmental conditions (1987-2008). Overall, our biological dataset gathered 14,367 occurrences of copepod species in the Mediterranean Sea.

Taxonomic classification ranging from order to genus was also added to the functional traits for each species, using the taxonomic standards used by Razouls et al. (2005-2015), which are those of Boxshall & Halsey (2004). In total, we considered 191 copepod species (Table 1), divided into 4 orders, 19 superfamilies, 34 families, and 70 genera (See Suppl. information for the full data table). Relying on independent basin-wide studies, this list does incorporate the most representative species of the Mediterranean copepod communities, both in terms of abundances and presences (Siokou-Frangou et al., 2010; Nowaczyk et al., 2011; Mazzocchi et al., 2014).

We completed our specific database with the description of 6 functional traits. They were chosen to cover various types and ecological functions (Fig.1 of Litchman et al., 2013), they are known to be ecologically meaningful (Kiørboe & Sabatini, 1994; Kiørboe, 2011; Kiørboe et al., 2015), and they are commonly used for zooplankton (Barnett et al., 2007; Pomerleau et al., 2015):

- two morphological traits related to all ecological functions: minimum and maximum body (cephalothorax) length of adults (mm);
- one physiological trait defining the species’ trophic group, defined into 5 classes: Carnivore, Omnivore-Carnivore, Omnivore, Omnivore-Herbivore, Omnivore-Detritivore;
- one behavioural trait depicting feeding strategy, defined into 4 classes (following Kiørboe, 2011): Active Ambush feeding, Cruise feeding, Filter feeding, Mixed feeding (for species that can switch between the 3 strategies);
- one life history trait related to reproduction: egg spawning strategy, defined into 2 classes: Broadcast-spawner (females release the eggs directly in the water) and Sac-spawner (eggs are carried by the females within sacs prior to hatching);
- one behavioural trait related to diel vertical migration (DVM) behaviour, defined into 4 classes according to the intensity of the observed migration: non migrant, weak migrant (DVM occurs within tens of metres), strong migrant (over several hundreds of metres), and reverse migrant (for species that migrate deeper at night).

Additionally, to discuss the potential role of each functional group in the pelagic ecosystem, the species' preferential habitat layer was informed. Doing so will allow us to estimate whether the function is carried on at several depth intervals, or if a functional group is linked to processes that occur in deeper layers, such as the recycling of organic matter. Vertical distribution preference was defined into 5 classes: epipelagic (0-200 m), epimesopelagic (0-1000 m), epibathypelagic (0-4000 m), mesopelagic (200-1000 m), mesobathypelagic (200-4000 m). The body sizes were obtained for all species from Razouls et al. (2005-2015), whereas the other traits were obtained from an extensive literature review (Supplementary Material S4). When available, trophic groups and feeding type were extrapolated to the genera since this trait is largely conserved within a genus (Kouwenberg, 1994). In total, we were able to determine at least 5 of the 7 functional traits for 171 species. Trophic group could be determined for 174 species (using 36 references), feeding type for 130 species (using 32 references), spawning strategy for 136 species (using 34 references), DVM behaviour for 125 species (using 9 references) and vertical distribution preference for 187 species (using 11 references). The functional trait database for the 191 most representative species of Mediterranean copepods, including references, is available as Supplementary Table S2, and can be downloaded from the PANGAEA database (www.pangaea.de).

**S2 - Identifying functional groups from the functional trait using MCA and hierarchical clustering**

In order to describe the functional traits of Mediterranean copepods and to identify functional groups, we performed a Multiple Correspondence Analysis (MCA) on the trait database to define the functional space. MCA is an ordination method in reduced space for the multivariate analysis of categorical variables (Husson et al., 2010). The computation of the MCA functional space was performed on 4 traits: class of maximum body length, trophism, feeding type, and spawning strategy. Indeed, the minimum body length (being highly correlated to the maximum body length, R2 = 0.866, n = 191) was discarded to avoid giving more weight to size compared to the other traits. DVM behaviour was not taken into account for two reasons. On one hand it tends to be very plastic for most species, meaning that it is known to greatly vary according to the studied oceanic basin, the environmental fluctuations and species’ ontogeny (Hays, 2003). On the other hand, a preliminary MCA incorporating this trait showed it had no impact in the definition of functional groups.

Since MCA required categorical variables and performs a correspondence analysis (CA) on the corresponding Burt table, maximum body length was transformed into categorical a variable and trophism was converted into four binary variables to correctly represent mixed trophic regimes in the analysis (Carnivore, Omnivore, Herbivore, Detritivore). The maximum body length was converted into a categorical variable consisting in 4 size classes, which were identified on the basis of a cluster analysis: Size_1 from 0.50 to 1.80 mm, Size_2 from 1.89 to 2.85 mm, Size_3 from 3.00 to 5.70 mm, and Size_4 from 6.10-11.0 mm. This allowed determining size classes that were adapted to our set of species (i.e. the Mediterranean planktonic ecosystems are dominated by small size classes; Siokou-Frangou et al., 2010). Neither feeding type nor spawning strategy needed transformation. For a better representation of the functional space, only the 99 species whose those 4 traits were fully defined were used for the computation of the MCA space, but the 92 other species were used as supplementary objects to allow their representation in the MCA space (Husson et al., 2010). Similarly, DVM behaviour (non migrant, weak migrant, strong migrant, reverse migrant) and binary vertical layers (epipelagic, mesopelagic, bathypelagic) were used as supplementary variables.

Using these 4 binary traits (trophic regime, 2 classes each) and 3 qualitative functional traits (size classes, feeding type, and spawning strategy, with 4, 4 and 2 categories, respectively) 11 dimensions were computed by the MCA (= 18 - 7, total number of categories minus number of traits). Only the first 4 dimensions were significant according to the Kaiser-Guttman criterion (eigenvalues higher than the average) and represented 29.52 %, 18.01 %, 13.32 % and 9.92 % of the variance, respectively (Fig. S1). Feeding, spawning strategy and trophism contributed to the MCA1 the most. This first axis opposed sac-spawning carnivores, employing active ambush feeding or cruise feeding strategies, from broadcaster omnivores and herbivores with filter or mixed feeding behaviour. Size class and detritivory mainly contributed to the MCA2. This second axis distinguished detritivores and small species (Size_1) from larger species (Size_2, Size_3, Size_4). Feeding and size classes mainly contributed to the third (small active ambush and mixed feeders vs. large cruise and filter feeders) and fourth dimensions. DVM and vertical habitat layers did not structure the MCA space significantly, confirming that they should not be taken into account for calculating the functional space. Indeed, a preliminary MCA using the 6 traits leaded to similar results, but with a lower rate of explained variance along the first axes.

Using the species’ coordinates along the four axes (explaining 70.77 % of the total variance), the Euclidean distance among the 191 species in the MCA space was computed and hierarchical agglomerative clustering was performed on this distance matrix, with Ward’s aggregation method (Husson et al., 2010, Legendre & Legendre, 2012). This synoptic method was chosen to identify large groups of species sharing similar traits (i.e., close in the MCA functional space). Depending on the cutting level, 2, 3, or 6 clusters could be identified (Fig. 1). The first level distinguished species according to their trophic group (carnivore vs. non-carnivores). Among non-carnivorous species, the second level discriminates broadcasters vs. sac-spawners. Then, each of these groups can be divided into two subgroups with different size and/or feeding type. Since higher cut levels could not be clearly related to functional traits, 6 functional groups were retained (Table 1, Table 2). The functional group of each species was then reported on the MCA biplots (Fig. S1). Similar groups were obtained when using another clustering method (K-means) or accounting for DVM and vertical layers, thus confirming the robustness of the results.

All statistical analyses were performed with R version 3.2.1 (R Core Team, 2015). MCA was performed using the *FactoMineR* package version 1.28 (Lê et al., 2008; Husson et al., 2009).

**References**

Barnett, A. J., Finlay, K. and Beisner, B. E. (2007) Functional diversity of crustacean zooplankton communities: towards a trait-based classification. *Freshw. Biol*., 52, 796-813.

Boxshall, G.A. & Halsey, S.H. (2004) An introduction to copepod diversity. The Ray Society, (Part 1): pp. I-XV, 1-421. (Partl.II): pp. V-VII, 422-966.

Husson, F., Josse, J., Lê, S., Mazet, J. (2009) FactoMineR: Multivariate Exploratory Data Analysis and Data Mining with R. R package version 1.12, URL http://factominer.free.fr.

Lê, S., Josse, J., Husson, F. (2008) FactoMineR: An R Package for Multivariate Analysis. *J. Stat. Software*, 25(1), 1-18.

Hays, G. C. (2003) A review of the adaptive significance and ecosystem consequences of zooplankton diel vertical migrations. *Hydrobiologia*, 503, 163-170.

Husson, F., Lê, S. and Pagès, J. (2010) Exploratory Multivariate Analysis by Example Using R. Computer Science and Data analysis Series. Chapman & Hall/CRC, London.

Kiørboe, T. (2011) How zooplankton feed: mechanisms, traits and trade-offs. *Biol. Rev.,* 86, 311-339.

Kiørboe, T., Ceballos, S., and Thygesen, U. H. (2015) Interrelations between senescence, life history traits, and behaviour in planktonic copepods. *Ecology*, 96, 2225-2235.

Kouwenberg, J.H.M. (1994) Copepod Distribution in Relation to Seasonal Hydrographics and Spatial Structure in the North-western Mediterranean (Golfe du Lion). *Estuarine Coastal Shelf Sci.,* 38: 69-90.

Legendre, P. and Legendre, L. (2012) Numerical Ecology, 3rd English Edition. Developments in Environmental Modelling, Vol. 24. Elsevier Science BV, Amsterdam. xiv + 990 pp.

Litchman, E., Ohman, M. D. and Kiørboe T. (2013) Trait-based approaches to zooplankton communities*. J. Plankton Res.*, 35, 473-484.

Mazzocchi, M. G., Siokou, I., Tirelli, V., Bandelj, V., Fernandez de Puelles, M., Ak Örek, Y. de Olazabal, A., Gubanova, A., Kress, N., Protopapa, M., Solidoro, C., Taglialatela, S., and Terbiyik Kurt, T. (2014) Regional and seasonal characteristics of epipelagic mesozooplankton in the Mediterranean Sea based on an artificial neural network analysis. *J. Mar. Sys.*, 135, 64-80.

Nowaczyk, A., Carlotti, F., Thibault-Botha, D. and Pagano, M. (2011) Distribution of epipelagic metazooplankton across the Mediterranean Sea during the summer BOUM cruise. *Biogeosc.*, 8, 2159-2177.

Pomerleau, C., Sastri, A. R., and Beisner, B. E. (2015) Evaluation of functional trait diversity for marine zooplankton communities in the Northeast subarctic Pacific Ocean. *J. Plankton Res.,* 37(4): 712-726.

R Core Team (2015). R: A language and environment for statistical computing. R Foundation for Statistical Computing, Vienna, Austria. URL http://www.R-project.org/

Razouls, C., de Bovée, F., Kouwenberg, J. and Desreumaux, N. (2005-2015) Diversity and Geographic Distribution of Marine Planktonic Copepods. Available at http://copepodes.obs-banyuls.fr/en [Accessed May 26, 2015].

Sameoto, D., P.H. Wiebe, J. Runge, L. Postel, J. Dunn, C. Miller and S. Coombs. (2000) Collecting zooplankton. In: Harris, R.P., P.H. Wiebe, J. Lenz, H.R. Skjoldal and M. Huntley (Eds), ICES Zooplankton Methodology Manual. Academic Press, London/San Diego: 55-81.

Siokou-Frangou, I., Christaki, U., Mazzocchi, M. G., Montresor, M., Ribera d’Alcalà, M., Vaqué, D., and Zingone, A. (2010) Plankton in the open Mediterranean Sea: a review. *Biogeosc.*, 7, 1543-1586.

Turner, J.T. (2004) The importance of small planktonic copepods and their roles in pelagic marine food webs. *Zool. Studies*, 43, 255-266.

UNESCO (1968) Zooplankton Sampling Monographs on Oceanographic Methodology. N. 2. UNESCO Press, Paris: 174 pp.

**S3 - List of the references used to list the most commonly-sampled species of Mediterranean copepods**

Carlotti, F. (2010) Mesozooplankton biomass and taxonomic distribution across the Mediterranean sea during the PROSOPE survey (PROSOPE). Proceedings from the 2010 AGU Ocean Sciences Meeting; 2010: American Geophysical Union, 2000 Florida Ave., N. W. Washington DC 20009 USA.

Gaudy, R., Youssara, F., Diaz, F., and Raimbault, P. (2003) Biomass, metabolism and nutrition of zooplankton in the Gulf of Lions (NW Mediterranean), *Oceanol. Acta*, 26(4), 357-372.

Mazzocchi, M., Christou, E., Fragopoulu, N., and Siokou-Frangou, I. (1997) Mesozooplankton distribution from Sicily to Cyprus (Eastern Mediterranean). 1. General aspects. *Oceanol. Acta*, 20(3), 521-535.

Mazzocchi, M.G., Nervegna, D., D'Elia, G., Di Capua, I., Aguzzi, L., and Boldrin, A. (2003) Spring mesozooplankton communities in the epipelagic Ionian Sea in relation to the Eastern Mediterranean Transient. *J. Geophys. Res.: Oceans*, 108, 8114, doi:10.1029/2002JC001640, C9.

Plounevez, S. and Champalbert, G. (2000) Diet, feeding behaviour and trophic activity of the anchovy (*Engraulis encrasicolus L.*) in the Gulf of Lions (Mediterranean Sea). *Oceanol. acta*, 23(2), 175-192.

Ramfos, A., Isari, S., Somarakis, S., Georgopoulos, D., Koutsikopoulos, C., and Fragopoulu, N. (2006) Mesozooplankton community structure in offshore and coastal waters of the Ionian Sea (eastern Mediterranean) during mixed and stratified conditions. *Mar. Biol.*, 150(1), 29-44.

Siokou-Frangou, I., Christou, E., Fragopoulu, N., and Mazzocchi, M.G. (1997) Mesozooplankton distribution from Sicily to Cyprus (Eastern Mediterranean). 2. Copepod assemblages. *Oceanol. Acta,* 20(3), 537-548.

Siokou-Frangou, I., Pancucci-Papadopoulou, M., and Christou, E. (1994) Sur la repartition du zooplankton superficiel des mers entourant la Grèce (Printemps 1987). *Biol Gallo-Hell*., 21, 313-330.

Siokou-Frangou, I., Pancucci-Papadopoulou, M., and Kouyoufas, P. (1990) Etude de la repartition du zooplancton dans les mers Egée et Ionienne. *Rapp. PV Reun. Comm. Int. Explor. Sci. Mer. Mediterr.,* 32, 221.

Siokou-Frangou, I. and Pancucci-Papadopoulou, M.A. (1996) Spatial patterns of copepods in the Aegean Sea. 6th International Conference on Copepoda, Oldenburg. Oral presentation. Book of abstracts p.100.

Thibault, D., Gaudy, R., and Le Fèvre, J. (1994) Zooplankton biomass, feeding and metabolism in a geostrophic frontal area (Almeria-Oran Front, western Mediterranean). Significance to pelagic food webs. *J. Mar. Sys.*, 5(3), 297-311.

Youssara, F. and Gaudy, R. (2001) Variations of zooplankton in the frontal area of the Alboran sea (Mediterranean sea) in winter 1997. *Oceanol. acta*, 24(4), 361-376.

Zervoudaki, S., Christou, E., Nielsen, T., Siokou-Frangou, I., Assimakopoulou, G., Giannakourou, A., Maar, M., Pagou, K., Krasakopoulou, E., Christaki, U. and Moraitou-Apostolopoulou, M. (2007) The importance of small-sized copepods in a frontal area of the Aegean Sea. *J. Plankton Res.*, 29(4), 317-338.

**S4 - List of the references used to inform the functional traits database of the 191 most representative species of Mediterranean copepods**

***A) Minimum and Maximum body lengths***

**A1** Razouls C., de Bovée F., Kouwenberg J. and Desreumaux N. (2005-2015). Diversity and Geographic Distribution of Marine Planktonic Copepods. Available at http://copepodes.obs-banyuls.fr/en

***B) Trophic regime***

**B1** Alldredge A.L. (1972) Abandoned larvacean houses: a unique food source in the pelagic environment. *Science*, 177(4052), 885-887.

**B2** Arashkevich Y.G. (1969) The food and feeding of copepods in the northwestern Pacific. *Oceanology*, 9(61), 695-709.

**B3** Arcos F. and Fleminger A. (1986) Distribution of filter-feeding calanoid copepods in the eastern equatorial Pacific. *California Cooperative Oceanic Fisheries Investigations Reports,* 27, 170-187.

**B4** Swadling K.M., Slotwinski A., Davies C., Beard J., McKinnon A.D., Coman F., Murphy N., Tonks M., Rochester W., Conway D.V.P., Hosie G.W. and Richardson A.J. (2013) Australian Marine Zooplankton: a taxonomic guide and atlas. Version 1.0 February 2013. Available online at http://www.imas.utas.edu.au/zooplankton

**B5** Boxshall G.A. and Halsey S.H. (2004) An introduction to copepod diversity. Ray Society.

**B6** Broglio E., Saiz E., Calbet A., Trepat I., Alcaraz M. (2004) Trophic impact and prey selection by crustacean zooplankton on the microbial communities of an oligotrophic coastal area (NW Mediterranean Sea). *Aquatic microbial ecology*, 35(1), 65-78.

**B7** Calbet A., Carlotti F. and Gaudy R. (2007) The feeding ecology of the copepod *Centropages typicus* (Kröyer). *Progress in Oceanography*, 72(2), 137-150.

**B8** Castellani C., Irigoien X., Mayor D.J., Harris R.P. and Wilson D. (2008) Feeding of *Calanus finmarchicus* and *Oithona similis* on the microplankton assemblage in the Irminger Sea, North Atlantic. *Journal of Plankton Research*, 30(10), 1095-1116.

**B9** El Shabrawy G.M. and Belmonte G. (2004) Abundance and affirmation of *Paracartia latisetosa* (Copepoda, Calanoida) in the inland lake Qarun (Egypt). *Thalassia Salentina*, 27, 151-160.

**B10** Gonzalez H.E. and Smetacek V. (1994) The possible role of the cyclopoid copepod Oithona in retarding vertical flux of zooplankton faecal material. *Marine Ecology Progress Series*, 113(3), 233-246.

**B11** Gowing M.M. and Wishner K.F. (1986) Trophic relationships of deep-sea calanoid copepods from the benthic boundary layer of the Santa Catalina Basin, California. *Deep Sea Research Part A Oceanographic Research Papers*, 33(7), 939-961.

**B12** Gowing M.M. and Wishner K.F. (1992) Feeding ecology of benthopelagic zooplankton on an eastern tropical Pacific seamount. *Marine Biology*, 112(3), 451-467.

**B13** Heron A. (1973) A specialized predator-prey relationship between the copepod *Sapphirina angusta* and the pelagic tunicate *Thalia democratica*. *Journal of the Marine Biological Association of the United Kingdom*, 53(02), 429-435.

**B14** Kosobokova K., Hirche H.-J., Scherzinger T. (2002) Feeding ecology of *Spinocalanus antarcticus*, a mesopelagic copepod with a looped gut. *Marine Biology*, 141(3), 503-511.

**B15** Kouwenberg J.H.M. (1994) Copepod distribution in relation to seasonal hydrographics and spatial structure in the Northwestern Mediterranean (Golfe du Lion). *Estuarine, Coastal and Shelf Science*, 38, 69-90.

**B16** Maar M., Visser A., Nielsen T.G., Stips A. and Saito H. (2006) Turbulence and feeding behaviour affect the vertical distributions of *Oithona similis* and *Microsetella norvegica*. *Marine Ecology Progress Series,* 313, 157-172.

**B17** Mauchline, J. (1998) The biology of calanoid copepods. *Adv. Mar. Biol.* 33, Eds: Blaxter, J.H.S., Southward, A.J., and Tyler, P.A., Academic press. 463 pp.

**B18** Moore P.A., Fields D.M. and Yen J. (1999) Physical constraints of chemoreception in foraging copepods. *Limnology and Oceanography*, 44(1), 166-177.

**B19** Nishida S. and Ohtsuka S. (1996) Specialized feeding mechanism in the pelagic copepod genus Heterorhabdus (Calanoida: Heterorhabdidae), with special reference to the mandibular tooth and labral glands. *Marine Biology*, 126(4), 619-632.

**B20** Nishida S. and Ohtsuka S. (1997) Ultrastructure of the mouthpart sensory setae in mesopelagic copepods of the family Scolecitrichidae. *Plankton Biology and Ecology*, 44, 81-90.

**B21** Ohtsuka S. and Onbé T. (1989) Evidence of selective feeding on larvaceans by the pelagic copepod *Candacia bipinnata* (Calanoida: Candaciidae). *Journal of Plankton Research*, 11(4), 869-872.

**B22** Ohtsuka S. and Onbé T. (1991) Relationship between mouthpart structures and *in situ* feeding habits of species of the family Pontellidae (Copepoda: Calanoida). *Marine Biology,* 111(2), 213-225.

**B23** Ohtsuka S., Kubo N., Okada M. and Gushima K. (1993) Attachment and feeding of pelagic copepods on larvacean houses. *Journal of Oceanography*, 49(1), 115-120.

**B24** Ohtsuka S., Böttger-Schnack R., Okada M. and Onbé T. (1996) *In situ* feeding habits of Oncaea (Copepoda: Poecilostomatoida) from the upper 250 m of the central Red Sea, with special reference to consumption of appendicularian houses. *Bulletin of Plankton Society of Japan,* 43(2), 89-105.

**B25** Olsen E.M., Jørstad T. and Kaartvedt S. (2000) The feeding strategies of two large marine copepods. *Journal of Plankton Research*, 22(8), 1513-1528.

**B26** Razouls C., de Bovée F., Kouwenberg J. and Desreumaux N. (2005-2015) Diversity and Geographic Distribution of Marine Planktonic Copepods. Available at http://copepodes.obs-banyuls.fr/en

**B27** Calbet A. and Saiz E. (2005) The ciliate-copepod link in marine ecosystems. *Aquatic Microbial Ecology*, 38(2), 157-167.

**B28** Steinberg D.K., Silver M.W., Pilskaln C.H., Coale S.L. and Paduan J.B. (1994) Midwater zooplankton communities on pelagic detritus (giant larvacean houses) in Monterey Bay, California. *Limnology and Oceanography*, 39(7), 1606-1620.

**B29** Takahashi K., Ichikawa T., Saito H., Kakehi S., Sugimoto Y., Hidaka K. and Hamasaki K. (2013) Sapphirinid copepods as predators of doliolids: Their role in doliolid mortality and sinking flux. *Limnology and Oceanography*, 58(6), 1972-1984.

**B30** Turner J.T., Tester P.A. and Conley W.J. (1984) Zooplankton feeding ecology: predation by the marine cyclopoid copepod *Corycaeus amazonicus* F. Dahl upon natural prey. *Journal of Experimental Marine Biology and Ecology*, 84(2), 191-202.

**B31** Turner J.T. (1986) Zooplankton feeding ecology: contents of fecal pellets of the cyclopoid copepods *Oncaea venusta*, *Corycaeus amazonicus*, *Oithona plumifera*, and *O. simplex* from the northern Gulf of Mexico. *Marine Ecology*, 7(4), 289-302.

**B32** Verheye H., Hagen W., Auel H., Ekau W., Loick N., Rheenen I., Wencke P. and Jones S. (2005) Life strategies, energetics and growth characteristics of *Calanoides carinatus* (Copepoda) in the Angola-Benguela frontal region. *African Journal of Marine Science*, 27(3), 641-651.

**B33** Wickstead J.H. (1959) A predatory copepod. *The Journal of Animal Ecology,* 28(1), 69-72.

**B34** Wickstead J.H. (1962) Food and feeding in pelagic copepods. *Proceedings of the Zoological Society of London*, 139(4), 545-555.

**B35** Yamaguchi A., Watanabe Y., Ishida H., Harimoto T., Furusawa K., Suzuki S., Ishizaka J., Ikeda T. and Takahashi M.M. (2002) Community and trophic structures of pelagic copepods down to greater depths in the western subarctic Pacific (WEST-COSMIC). *Deep Sea Research Part I: Oceanographic Research Papers,* 49(6), 1007-1025.

**B36** Yen J. (1987) Predation by a carnivorous marine copepod, *Euchaeta norvegica* Boeck, on eggs and larvae of the North Atlantic cod *Gadus morhua L*. *Journal of Experimental Marine Biology and Ecology*, 112(3), 283-296.

***C) Feeding type***

**C1** Arashkevich Y.G. (1969) The food and feeding of copepods in the northwestern Pacific. *Oceanology*, 9(61), 695-709.

**C2** Arcos F. and Fleminger A. (1986) Distribution of filter-feeding calanoid copepods in the eastern equatorial Pacific. *California Cooperative Oceanic Fisheries Investigations Reports,* 27, 170-187.

**C3** Swadling K.M., Slotwinski A., Davies C., Beard J., McKinnon A.D., Coman F., Murphy N., Tonks M., Rochester W., Conway D.V.P., Hosie G.W. and Richardson A.J. (2013) Australian Marine Zooplankton: a taxonomic guide and atlas. Version 1.0 February 2013. Available online at http://www.imas.utas.edu.au/zooplankton

**C4** Barton A.D., Pershing A.J., Litchman E., Record N.R., Edwards K.F., Finkel Z.V., Kiørboe T. and Ward B.A. (2013) The biogeography of marine plankton traits. *Ecology Letters*, 16: 522–534.

**C5** Calbet A., Carlotti F. and Gaudy R. (2007) The feeding ecology of the copepod *Centropages typicus* (Kröyer). *Progress in Oceanography,* 72(2), 137-150.

**C6** Go Y.-B., Oh B.-C. and Terazaki M. (1998) Feeding behavior of the poecilostomatoid copepods *Oncaea spp*. on chaetognaths. *Journal of Marine Systems,* 15(1), 475-82.

**C7** Gophen M. and Harris R. (1981) Visual predation by a marine cyclopoid copepod, *Corycaeus anglicus*. *Journal of the Marine Biological Association of the United Kingdom,* 61(02), 391-399.

**C8** Greene C.H. and Landry M.R. (1985) Patterns of prey selection in the cruising calanoid predator *Euchaeta elongata*. *Ecology,* 66, 1408–1416.

**C9** Harding G. (1974) The food of deep-sea copepods. *Journal of the Marine Biological Association of the United Kingdom,* 54(01), 141-155.

**C10** Heron A. (1973) A specialized predator-prey relationship between the copepod *Sapphirina angusta* and the pelagic tunicate *Thalia democratica*. *Journal of the Marine Biological Association of the United Kingdom,* 53(02), 429-435.

**C11** Kiørboe T. and Saiz E. (1995) Planktivorous feeding in calm and turbulent environments, with emphasis on copepods. *Marine Ecology Progress Series,* 122(1-3), 135-145.

**C12** Kiørboe T., Andersen A., Langlois V.J., Jakobsen H.H. and Bohr T. (2009) Mechanisms and feasibility of prey capture in ambush-feeding zooplankton. *Proceedings of the National Academy of Sciences,* 106(30), 12394-12399.

**C13** Kiørboe T. (2011) How zooplankton feed: mechanisms, traits and trade-offs. *Biological Reviews*, 86(2), 311-339.

**C14** Kosobokova K., Hirche H.-J. and Scherzinger T. (2002) Feeding ecology of *Spinocalanus antarcticus*, a mesopelagic copepod with a looped gut. *Marine Biology,* 141(3), 503-511.

**C15** Kouwenberg J.H.M. (1994) Copepod distribution in relation to seasonal hydrographics and spatial structure in the Northwestern Mediterranean (Golfe du Lion). *Estuarine, Coastal and Shelf Science,* 38, 69-90.

**C16** Landry M., Lehner-Fournier J. and Fagerness V. (1985) Predatory feeding behavior of the marine cyclopoid copepod *Corycaeus anglicus*. *Marine Biology,* 85(2), 163-169.

**C17** Maar M., Visser A., Nielsen T.G., Stips A. and Saito H. (2006) Turbulence and feeding behaviour affect the vertical distributions of *Oithona similis* and *Microsetella norvegica*. *Marine Ecology Progress Series,* 313, 157-172.

**C18** Mauchline, J. (1998) The biology of calanoid copepods. *Adv. Mar. Biol.* 33, Eds: Blaxter, J.H.S., Southward, A.J., and Tyler, P.A., Academic press. 463 pp.

**C19** Moore P.A., Fields D.M. and Yen J. (1999) Physical constraints of chemoreception in foraging copepods. *Limnology and Oceanography,* 44(1), 166-177.

**C20** Ohtsuka S. and Onbé T. (1991) Relationship between mouthpart structures and in situ feeding habits of species of the family Pontellidae (Copepoda: Calanoida). *Marine Biology,* 111(2), 213-225.

**C21** Olsen E.M., Jørstad T. and Kaartvedt S. (2000) The feeding strategies of two large marine copepods. *Journal of Plankton Research,* 22(8), 1513-1528.

**C22** Paffenhöfer G.-A. and Knowles S.C. (1980) Omnivorousness in marine planktonic copepods. *Journal of Plankton Research*, 2(4), 355-365.

**C23** Paffenhöfer G.-A. (1993) On the ecology of marine cyclopoid copepods (Crustacea, Copepoda). *Journal of Plankton Research,* 15(1), 37-55.

**C24** Paffenhöfer G.-A. (1998) On the relation of structure, perception and activity in marine planktonic copepods. *Journal of Marine Systems,* 15(1), 457-473.

**C25** Paffenhöfer G.-A. and Mazzocchi M. (2002) On some aspects of the behaviour of *Oithona plumifera* (Copepoda: Cyclopoida). *Journal of Plankton Research,* 24(2), 129-135.

**C26** Razouls C., de Bovée F., Kouwenberg J. and Desreumaux N. (2005-2015) Diversity and Geographic Distribution of Marine Planktonic Copepods. Available at http://copepodes.obs-banyuls.fr/en

**C27** Siokou-Frangou I., Christaki U., Mazzocchi M., Montresor M., d'Alcala M.R., Vaqué D. and Zingone, A. (2010) Plankton in the open Mediterranean Sea: a review. *Biogeosciences*, 7(5), 1543-1586.

**C28** Takahashi K., Ichikawa T., Saito H., Kakehi S., Sugimoto Y., Hidaka K. and Hamasaki K. (2013) Sapphirinid copepods as predators of doliolids: Their role in doliolid mortality and sinking flux. *Limnology and Oceanography*, 58(6), 1972-1984.

**C29** Tiselius P. and Jonsson P.R. (1990) Foraging behavior of 6 calanoid copepods - Observations and hydrodynamic analysis. *Marine Ecology Progress Series,* 66(1-2), 23-33.

**C30** Wickstead J.H. (1962) Food and feeding in pelagic copepods. *Proceedings of the Zoological Society of London*, 139(4), 545-555.

**C31** Yamaguchi A., Watanabe Y., Ishida H., Harimoto T., Furusawa K., Suzuki S., Ishizaka J., Ikeda T. and Takahashi M.M. (2002) Community and trophic structures of pelagic copepods down to greater depths in the western subarctic Pacific (WEST-COSMIC). *Deep Sea Research Part I: Oceanographic Research Papers,* 49(6), 1007-1025.

**C32** Yen J. (1987) Predation by a carnivorous marine copepod, *Euchaeta norvegica* Boeck, on eggs and larvae of the North Atlantic cod *Gadus morhua L*. *Journal of Experimental Marine Biology and Ecology*, 112(3), 283-296.

***D) Diel Vertical Migration***

**D1** Andersen V., Gubanova A., Nival P. and Ruellet T. (2001) Zooplankton community during the transition from spring bloom to oligotrophy in the open NW Mediterranean and effects of wind events. 2. Vertical distributions and migrations. *Journal of Plankton Research,* 23(3), 243-261.

**D2** Andersen V., Devey C., Gubanova A., Picheral M., Melnikov V., Tsarin S. and Prieur, L. (2004) Vertical distributions of zooplankton across the Almeria-Oran frontal zone (Mediterranean Sea). *Journal of Plankton Research,* 26(3), 275-293.

**D3** Auel H. and Hagen W. (2002) Mesozooplankton community structure, abundance and biomass in the central Arctic Ocean. *Marine Biology,* 140(5), 1013-1021.

**D4** Böttger-Schnack R. (1990) Community structure and vertical distribution of cyclopoid copepods in the Red Sea. *Marine Biology,* 106(3), 473-485.

**D5** Böttger-Schnack R. (1996) Vertical structure of small metazoan plankton, especially non calanoid copepods. I. Deep Arabian Sea. *Journal of Plankton Research,* 18(7), 1073-1101.

**D6** Boucher J. (1984) Localization of zooplankton populations in the Ligurian marine front: role of ontogenic migration. *Deep Sea Research Part A Oceanographic Research Papers,* 31(5), 469-484.

**D7** Boxshall G. (1977) The depth distributions and community organization of the planktonic cyclopoids (Crustacea: Copepoda) of the Cape Verde Islands region. *Journal of the Marine Biological Association of the United Kingdom,* 57(02), 543-568.

**D8** Brugnano C., Granata A., Guglielmo L. and Zagami G. (2012) Spring diel vertical distribution of copepod abundances and diversity in the open Central Tyrrhenian Sea (Western Mediterranean). *Journal of Marine Systems,* 105, 207-220.

**D9** Chen Y-Q. (1986) The vertical distribution of some pelagic copepods in the eastern tropical Pacific. *CalCOFI Report*, 27, 205-227.

**D10** Furnestin M-L. (1960) Zooplancton du Golfe du Lion et de la côte orientale de Corse. *Revue des Travaux de l'Institut des Pêches maritimes,* 24(2), 153-252.

**D11** Hure J. and Scotto di Carlo B. (1974) New patterns of diurnal vertical migration of some deep-water copepods in the Tyrrhenian and Adriatic Seas. *Marine Biology,* 28(3), 179-184.

**D12** Irigoien X., Conway D.V. and Harris R.P. (2004) Flexible diel vertical migration behaviour of zooplankton in the Irish Sea. *Marine Ecology Progress Series,* 267, 85-97.

**D13** Kobari T., Shinada A. and Tsuda A. (2003) Functional roles of interzonal migrating mesozooplankton in the western subarctic Pacific. *Progress in Oceanography*, 57(3), 279-298.

**D14** Lo W., Shih C.-T. and Hwang J. (2004) Copepod assemblages and diel vertical migration in the East China Sea, north of Taiwan. *Crustaceana,* 77(8), 955-971.

**D15** Longhurst A. and Williams R. (1979) Materials for plankton modelling: vertical distribution of Atlantic zooplankton in summer. *Journal of Plankton Research,* 1(1), 1-28.

**D16** McGowan J.A. and Walker P.W. (1979) Structure in the copepod community of the North Pacific central gyre. *Ecological monographs,* 195-226.

**D17** Moraitou-Apostolopoulou M. (1971) Vertical distribution, diurnal and seasonal migration of copepods in Saronic Bay, Greece. *Marine Biology,* 9(2), 92-98.

**D18** Padmavati G., Haridas P., Nair K., Gopalakrishnan T., Shiney P. and Madhupratap M. (1998) Vertical distribution of mesozooplankton in the central and eastern Arabian Sea during the winter monsoon. *Journal of plankton Research,* 20(2), 343-354.

**D19** Roe H. (1972) The vertical distributions and diurnal migrations of Calanoid copepods collected on the SOND cruise, 1965 I. The total population and general discussion. *Journal of the Marine Biological Association of the United Kingdom,* 52(02), 277-314.

**D20** Roe H. (1972) The vertical distributions and diurnal migrations of Calanoid copepods collected on the SOND cruise, 1965. II. Systematic account: families Calanidae up to and including the Aetideidae. *Journal of the Marine Biological Association of the United Kingdom,* 52(02), 315-343.

**D21** Roe H. (1972) The vertical distributions and diurnal migrations of Calanoid copepods collected on the SOND cruise, 1965 III. Systematic account: families Euchaetidae up to and Including the Metridiidae. *Journal of the Marine Biological Association of the United Kingdom,* 52(03), 525-552.

**D22** Roe H. (1972) The vertical distributions and diurnal migrations of Calanoid copepods collected on the SOND cruise, 1965 IV. Systematic account of families Lucicutiidae to Candaciidae. The relative abundance of the numerically most important genera. *Journal of the Marine Biological Association of the United Kingdom,* 52(04), 1021-1044.

**D23** Roe H. (1974) Observations on the diurnal vertical migrations of an oceanic animal community. *Marine Biology,* 28(2), 99-113.

**D24** Roe H. (1984) The diel migrations and distributions within a mesopelagic community in the North East Atlantic. 4. The copepods. *Progress in Oceanography,* 13(3), 353-388.

**D25** Saraswathy M. (1982) Siphonostomes (Copepoda-Cyclopoida) from the Indian Ocean. *Journal of Plankton Research,* 4(3), 633-641.

**D26** Scotto di Carlo B., Ianora A., Fresi E. and Hure J. (1984) Vertical zonation patterns for Mediterranean copepods from the surface to 3000 m at a fixed station in the Tyrrhenian Sea. *Journal of Plankton Research,* 6(6), 1031-1056.

**D27** Siokou-Frangou I. (1996) Zooplankton annual cycle in a Mediterranean coastal area. *Journal of Plankton Research,* 18(2), 203-223.

**D28** Southward A. and Barrett R. (1983) Observations on the vertical distribution of zooplankton, including post-larval teleosts, off Plymouth in the presence of a thermocline and a chlorophyll-dense layer. *Journal of Plankton Research,* 5(4), 599-618.

**D29** Steinberg D.K., Cope J.S., Wilson S.E. and Kobari T. (2008) A comparison of mesopelagic mesozooplankton community structure in the subtropical and subarctic North Pacific Ocean. *Deep Sea Research Part II: Topical Studies in Oceanography,*  55(14), 1615-1635.

**D30** Vives F. (1978) Distribucion de la poblacion de copepodos en el Mediterraneo occidental. *Resultados Expediciones Cientificas del Buque Oceanografico Cornide de Saavedra.* Suppl. 7, 263-302.

**D31** Weikert H. and Trinkaus S. (1990) Vertical mesozooplankton abundance and distribution in the deep Eastern Mediterranean Sea SE of Crete. *Journal of Plankton Research,* 12(3), 601-628.

**D32** Williams R. (1988) Spatial heterogeneity and niche differentiation in oceanic zooplankton. *Hydrobiologia,* 167(1), 151-159.

**D33** Yamaguchi A., Watanabe Y., Ishida H., Harimoto T., Furusawa K., Suzuki S., Ishizaka J., Ikeda T. and Takahashi M.M. (2002) Community and trophic structures of pelagic copepods down to greater depths in the western subarctic Pacific (WEST-COSMIC). *Deep Sea Research Part I: Oceanographic Research Papers,* 49(6), 1007-1025.

**D34** Zalkina A. (1970) Vertical distribution and diurnal migration of some Cyclopoida (Copepoda) in the tropical region of the Pacific Ocean. *Marine Biology,* 5(4), 275-282.

***E) Spawning strategy***

**E1** Bunker A. and Hirst A. (2004) Fecundity of marine planktonic copepods: global rates and patterns in relation to chlorophyll a, temperature and body weight. *Marine Ecology-Progress Series,* 279,161-181.

**E2** Calbet A. and Agustí S. (1999) Latitudinal changes of copepod egg production rates in Atlantic waters: temperature and food availability as the main driving factors. *Marine Ecology-Progress Series,* 181, 155-162.

**E3** Hirst A. and Kiørboe T. (2002) Mortality of marine planktonic copepods: global rates and patterns. *Marine Ecology-Progress Series,* 230, 195-209.

**E4** Kiørboe T. and Sabatini M. (1994) Reproductive and life cycle strategies in egg-carrying cyclopoid and free-spawning calanoid copepods. *Journal of Plankton Research,* 16(10), 1353-1366.

**E5** Kiørboe T. and Sabatini M. (1995) Scaling of fecundity, growth and development in marine planktonic copepods. *Marine Ecology-Progress Series,* 120(1-3), 285-298.

**E6** Kosobokova K., Hirche H.-J. and Hopcroft R. (2007) Reproductive biology of deep-water calanoid copepods from the Arctic Ocean. *Marine Biology,* 151(3), 919-934.

**E7** Mauchline, J. (1998) The biology of calanoid copepods. *Adv. Mar. Biol.* 33, Eds: Blaxter, J.H.S., Southward, A.J., and Tyler, P.A., Academic press. 463 pp.

**E8** Niehoff B. (2007) Life history strategies in zooplankton communities: The significance of female gonad morphology and maturation types for the reproductive biology of marine calanoid copepods. *Progress in Oceanography,* 74(1), 1-47.

**E9** Saiz E. and Calbet A. (1999) On the free-spawning reproductive behaviour of the copepod *Clausocalanus lividus* (Frost and Fleminger 1968). *Journal of Plankton Research,* 21, 599-602.

***F) Vertical distribution***

**F1** Andersen V., Gubanova A., Nival P. and Ruellet T. (2001) Zooplankton community during the transition from spring bloom to oligotrophy in the open NW Mediterranean and effects of wind events. 2. Vertical distributions and migrations. *Journal of Plankton Research,* 23(3), 243-261.

**F2** Andersen V., Devey C., Gubanova A., Picheral M., Melnikov V., Tsarin S. and Prieur, L. (2004) Vertical distributions of zooplankton across the Almeria-Oran frontal zone (Mediterranean Sea). *Journal of Plankton Research,* 26(3), 275-93.

**F3** Böttger-Schnack R. and Schnack D. (1989) Vertical distribution and population structure of *Macrosetella gracilis* (Copepoda: Harpacticoida) in the Red Sea in relation to the occurrence of *Oscillatoria* (Trichodesmium) *spp.* (Cyanobacteria). *Marine Ecology Progress Series,* 52(1), 17-31.

**F4** Böttger-Schnack R. (1990) Community structure and vertical distribution of cyclopoid copepods in the Red Sea. *Marine Biology,* 106(3), 473-485.

**F5** Böttger-Schnack R. (1990) Vertical structure of small metazoan plankton, especially noncalanoid copepods. I. Deep Arabian Sea. *Journal of Plankton Research,* 18(7), 1073-1101.

**F6** Böttger-Schnack R. (1996) Vertical structure of small metazoan plankton, especially non-calanoid copepods. 2. Deep Eastern Mediterranean (Levantine sea). *Oceanolica Acta,* 20(2), 399-419.

**F7** Böttger-Schnack R., Hagen W. and Schnack-Schiel S. (2001) The microcopepod fauna in the Gulf of Aqaba, northern Red Sea: species diversity and distribution of Oncaeidae (Poecilostomatoida). *Journal of Plankton Research,* 23(9), 1029-1035.

**F8** Boxshall G.A. and Halsey S.H. (2004) An introduction to copepod diversity. Ray Society.

**F9** Brugnano C., Granata A., Guglielmo L. and Zagami G. (2012) Spring diel vertical distribution of copepod abundances and diversity in the open Central Tyrrhenian Sea (Western Mediterranean). *Journal of Marine Systems,* 105, 207-220.

**F10** Razouls C., de Bovée F., Kouwenberg J. and Desreumaux N. (2005-2015) Diversity and Geographic Distribution of Marine Planktonic Copepods. Available at http://copepodes.obs-banyuls.fr/en

**F11** Scotto di Carlo B., Ianora A., Fresi E. and Hure J. (1984) Vertical zonation patterns for Mediterranean copepods from the surface to 3000 m at a fixed station in the Tyrrhenian Sea. *Journal of Plankton Research,* 6(6), 1031-1056.

**Supplementary Tables:**

**Table S1.** Reviewed references for listing the most representative species of Mediterranean copepods. For each sampling cruise, the month and year of sampling are indicated, as well as the reference paper (see Suppl. Information S3) and the reference dataset, when available.

**Table S2.** Functional traits of the most representative copepod species of the Mediterranean Sea. Seven traits are described (see text): minimal and maximal body lengths (mm), trophic group, feeding type, spawning strategy, diel vertical migration behaviour, and vertical habitat. All the references used to compile this data set are indicated and listed in the Suppl. Information S4.

**Supplementary Figures:**

**Figure S1.** Position of the 191 copepod species in the functional space obtained from an MCA based on 4 functional traits: class of maximum body length (Size_1: 0.50 to 1.80 mm, Size_2: 1.89 to 2.85 mm, Size_3: 3.00 to 5.70 mm, Size_4: 6.10-11.0 mm), binary trophic group (Carnivore, Omnivore, Herbivore, Detritivore), feeding type (Active Ambush feeding, Cruise feeding, Filter feeding, Mixed feeding), and spawning strategy (Broadcast-spawner, Sac-spawner). The first 4 axes of the MCA were significant according to the Kaiser-Guttman criterion. The 99 species whose traits were used for the computation of the MCA space are indicated with triangle. The 92 supplementary species are represented by circle. Supplementary variables (DVM and vertical layers) are indicated in grey. Colours indicate the functional groups as identified from the hierarchical clustering (Fig. 1).
