# Supplementary figures and images for: Identifying copepod functional groups from species functional traits
Source: J Plankton Res. 2015 Nov 3;38(1):159–66. doi: 10.1093/plankt/fbv096 (PMC4722884; doi:10.1093/plankt/fbv096)

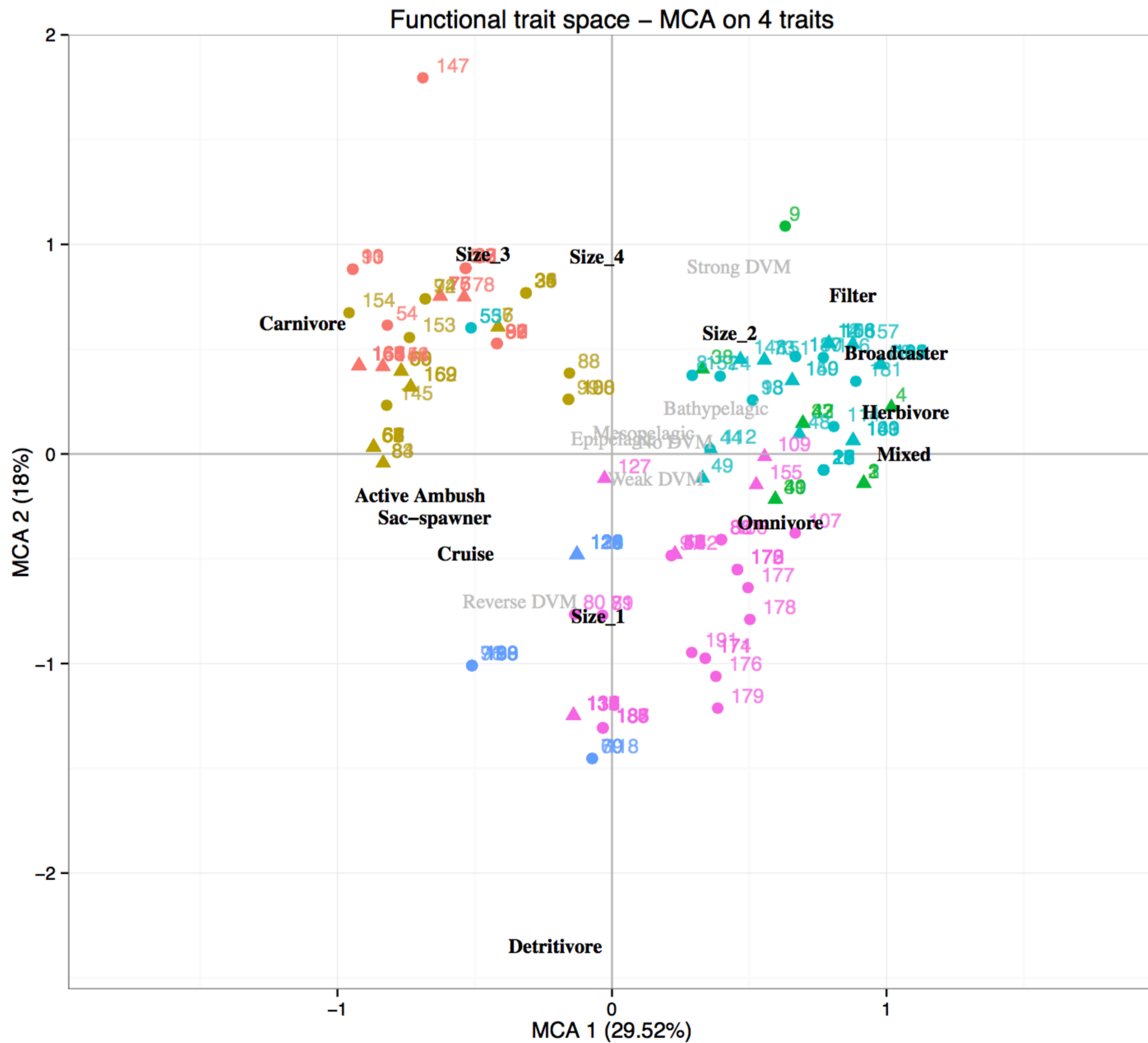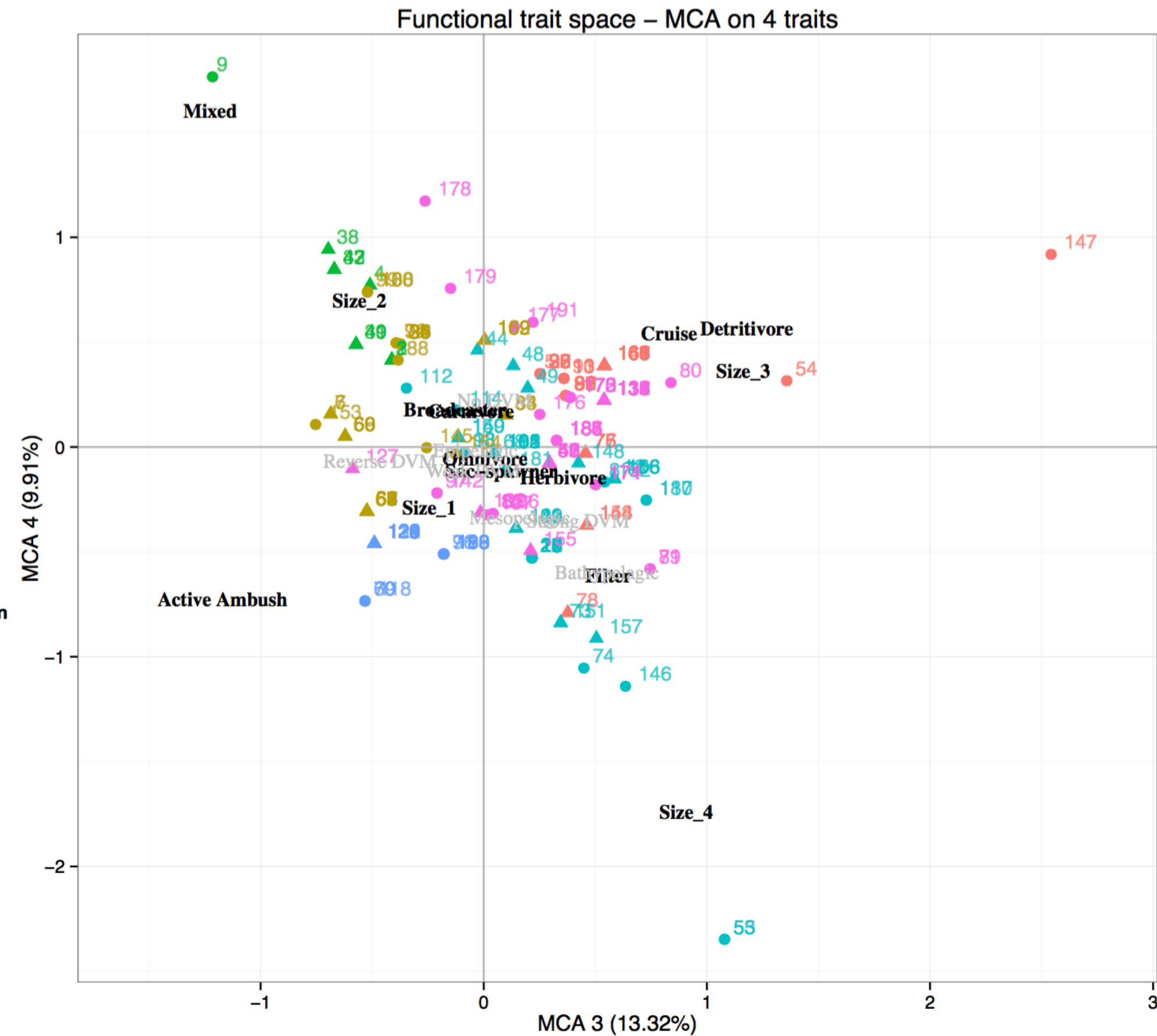

Supplement: Supplementary Data [file supp_fbv096_fbv096supp_fig1.pdf]
